# Insecticide susceptibility and dengue vector status of wild *Stegomyia albopicta* in a strategically important area of Assam, India

**DOI:** 10.1186/1756-3305-7-295

**Published:** 2014-07-01

**Authors:** Sunil Dhiman, Bipul Rabha, Kavita Yadav, Indra Baruah, Vijay veer

**Affiliations:** 1 Defence Research Laboratory, Tezpur Assam-784 001, India

**Keywords:** *St. albopicta*, Dengue, Northeast India, Insecticide resistance, VectorTest™ assay

## Abstract

**Background:**

Dengue vector control programmes are facing operational challenges due to resistance against commonly used insecticides throughout the endemic countries. Recently, there has been appreciable increase in the dengue cases in India, however, no recent data are available on susceptible status of dengue vectors. We have studied the susceptibility level of *St. albopicta* to commonly used insecticides in India. Adult mosquitoes were tested for the presence of dengue virus.

**Methods:**

*St. albopicta* larval bioassays were carried out to determine the lethal concentrations (LC_10_, LC_50_ and LC_99_) and the resistance ratios (RR_10_, RR_50_ and RR_99_) for temephos. Susceptibility to 4% DDT, 0.05% deltamethrin and 5% malathion was assessed following standard procedure. Knock-down times (KDT_10_, KDT_50_ and KDT_99_) were estimated and knock-down resistance ratios (KRR_10_, KRR_50_ and KRR_99_) were calculated. VectorTest™ dengue antigen assay was used to detect the dengue virus in the field collected mosquitoes.

**Results:**

In larval bioassays, the RR ranged from 1.4 (for RR_99_) to 1.7 (for RR_50_), which suggested that the tested *St. albopicta* were susceptible to temephos. There was no deviation among the lethal concentration data from linearity (r^2^ = 0.61). Adult *St. albopicta* mosquitoes were resistant to DDT, while fully susceptible to deltamethrin and malathion. The knock-down values (KDT_10_, KDT_50_ and KDT_99_) obtained for DDT displayed straight line in log-dose-probit analysis and follow linear regression model. The KRR_99_ for DDT was 4.9, which indicated a 4.9 folds increase in knock-down resistance to DDT. However, for malathion and deltamethrin, the KRR_99_ values were 1.6 and 1.5 respectively suggesting that mosquitoes were knock-down sensitive. None of the mosquito pool was dengue virus positive.

**Conclusion:**

*St. albopicta* showed resistance to DDT and reduced sensitivity to deltamethrin and malathion. This data on insecticide resistance could help public health authorities in India to design more effective vector control measures. More dengue vector specimens need to be scanned to identify the potential dengue vector.

## Background

Dengue fever is one of the major mosquito-borne viral infections in tropical and sub-tropical regions. In recent years, dengue transmission has increased predominantly in urban, semi-urban settings and has even extended to the rural settings and become a major public health concern at international level. The World Health Organisation (WHO) has estimated that over 2.5 billion people corresponding to over 40% of the world’s population are now at dengue risk [1]. In Southeast-Asian countries, the dengue outbreaks usually occur during the rainy season and are supported by *Stegomyia aegypti* mosquitoes in urban settings, while *Stegomyia albopicta* in the rural areas [2-4]. *St. albopicta* (previously *Aedes (Stg) albopictus*), popularly known as “Asian tiger mosquito”, feeds on humans in gardens, parks and bushes around human dwellings in the daytime and is very common in most of the Asian countries [5,6]. Since there is no specific vaccine and treatment available for dengue, the control mainly turns to the surveillance and control of vectors to reduce transmission in the endemic settings [7-10].

Dengue vector control strategies largely depend upon the use of larvicides in the breeding sites to target the vectors at larval level and space sprays to target the adult infective stages of the potential known dengue vectors. The space spraying is generally used during the outbreak situations, but may be used to target both larvae and adult stages at a time in an integrated approach. Inspite of tremendous anti-vector efforts, the vector control interventions are sometimes threatened due to the development and spread of insecticide resistance among the vector species [10,11]. Many recent studies have demonstrated either the development of resistance or decrease in the susceptibility to synthetic insecticides in *St. albopicta* and *St. aegypti* mosquitoes in many endemic countries [10-12], however, such data, especially on *St. albopicta* is very limited in India and only a few systematic studies have been carried out to establish its susceptibility status [13,14]. In India, recently dengue has dramatically spread to many areas where it was not reported earlier, owing to which there is urgent need to determine the susceptibility status of the prevalent dengue vector to the commonly used insecticides in the control programme. Further, active surveillance of infected mosquitoes to identify the vector may also be valuable in defining spatial and temporal risk of acquiring dengue infection in an area of interest. Currently, we have examined the distribution and insecticide susceptibility of both larval and adult *St. albopicta* in Tezpur military area of Northeast India. Tezpur military area is strategically important because it serves as a transit station for the Indian troops moving toward the forward areas along the Indo-China border. Keeping in view the fact that in 2013, the region has reported around 5,000 confirmed dengue cases, the present study was undertaken to establish the insecticide resistance status against the commonly used insectides and to identify the prevalent dengue vector in order to implement effective and sustainable arbovirus vector control measures in the region.

## Methods

### *Stegomyia* mosquito collection

*Stegomyia* mosquito aquatic stages (larvae and pupae) were collected in twenty one locations in the military station area (N 26° 39′ 10.2′′ to E 92° 47′ 33.1′′). The immature stages (F_0_ -generation) were collected from different types of breeding localities such as, storage tanks, tyres, tree holes, cut bamboos, construction sites, flower pots, plastic cups and drains available in the study area. For each sampling site, larvae or pupae from all the accessible breeding places were collected and transferred to the laboratory. *Stegomyia* mosquitoes are regularly maintained at the insectary of Defence Research Laboratory, Tezpur at controlled conditions (temperature- 25°C +/− 2°C; relative humidity 80% +/−10%) and the females are fed on rabbit blood to complete their gonotrophic cycle. The wild collected mosquitoes were reared to the adult stages in the laboratory and kept in an isolated room to prevent the inbreeding after identification to species level. The F1-generation of the collected mosquitoes was used for larval and adult bioassays. Further, the laboratory strain (LS) of the mosquitoes was used as reference strain to compare with the wild strain (WS).

### Larval bioassay

Susceptibility of larvae to the temephos (90.7% pure; Heranba Industries Ltd., Mumbai) was estimated using standard WHO bioassays [15]. The stock solution of 1 ppm and dilutions was prepared in 95% ethanol and stored at +4°C for use in the experiments. Bioassays were conducted using 20–25 third instar larvae (both WS and LS) in plastic cups filled with required concentration of insecticide solution and millipore water (Milli-Q, MA, USA) at room temperature (25°C ± 2°C). Four different concentrations (0.001, 0.005, 0.01 and 0.05 ppm) were used and each experiment was replicated at least three times. Each bioassay was accompanied by a control test to which only 95% ethanol was added in equal concentration. Corrected mortality was calculated after 24 h and the larvae were considered dead if they did not show any movement when induced with a glass rod.

### Adult susceptibility assay

The adult insecticide susceptibility assay was performed following standard WHO protocol and involved tarsal contact exposure to insecticide impregnated papers in the standard susceptibility test kit [16,17]. Susceptibility to 4% DDT, 0.05% deltamethrin and 5% malathion was assessed using insecticide pre-impregnated papers obtained from Universiti Sains Malaysia, Malaysia. Adult unfed females (3–5 days old) in the batches of 10–15 were exposed to the insecticide impregnated papers in the WHO tubes for 1 h and cumulative knock-down was recorded every 5 minutes. The surviving mosquitoes were transferred to the WHO holding tubes and fed on 5% sucrose solution. Mortality was scored after 24 h to grade the sensitivity status as per WHO recommendation [16].

### Dengue antigen assay

The adult *Stegomyia* mosquitoes flying or resting inside the barracks, washrooms and other places of high density, were collected to detect the dengue virus using VectorTest™ (Vector Test System Inc., CA) dengue antigen assay. The VectorTest™ assay is a highly specific and rapid immunochromatographic assay for qualitative detection of any of the four serotypes of dengue virus (DENV 1–4) in the infected mosquito [18]. The assays were performed following standard manufacturer’s instructions.

### Data analysis

Mortality obtained was corrected using Schneider-Orelli’s formula [19] and interpreted following WHO recommendation. Data from larval bioassays were analyzed using Line Log-Dose Probit software. Chi-square (χ^2^) test was used to estimate the goodness of fit, whereas linear regression (r^2^) was used to evaluate if the data deviate from the linearity. Lethal concentrations (LC_10_, LC_50_ and LC_99_) along with the slope were estimated at 95% confidence intervals (CIs). The resistance ratios (RR_10_, RR_50_ and RR_99_) were calculated by dividing the LC_10_, LC_50_ and LC_99_ values of WS with the similar values of LS (reference strain). A RR_99_ of < 2 corresponded to susceptible, whereas ≥ 2 was considered corresponding to the resistance. Knock-down time (KDT_10_, KDT_50_ and KDT_99_) and their 95% CIs were determined and used to calculate the knock-down resistance ratios (KRRs) (KRR_10_, KRR_50_ and KRR_99_) by dividing the KDT_10_, KDT_50_ and KDT_99_ values of WS with the similar values of LS. A KRR_99_ of < 2 implied susceptible, while ≥ 2 was considered implying knock-down resistance in WS *St. albopicta*.

## Results

The baseline LC_10_, LC_50_ and LC_99_ value obtained in the LS larval bioassay exhibited a straight-line relationship between the insecticide log dose and probit mortality (χ^2^ = 1.28; p = 0.6). Linear regression showed that there was no deviation among the LC data from linearity (r^2^ = 0.70) (Table 1). Similarly the LC_10_, LC_50_ and LC_99_ values observed in the WS also displayed in straight line probit relationship (χ^2^ = 2.34; p = 0.3) and the data did not deviate from linearity (r^2^ = 0.61). The RR values ranged from 1.4 (for RR_99_) to 1.7 (for RR_50_), which suggest that the tested populations were susceptible to temephos (Table 1).

**Table 1:** **Susceptibility of *St. albopicta *** **larvae to temephos**

**Values**	**WS (N = 293)**	**LS (N = 300)**
LC_10_ (95% CI)	0.0006 (0.0003-0.001)	0.0004 (0–0.001)
LC_50_ (95% CI)	0.0035 (0.0026-0.0046)	0.0021 (0.0004-0.0037)
LC_99_ (95% CI)	0.081 (0.045-0.20)	0.056 (0.026-0.60)
χ^2^ (p)	2.34 (0.3)	1.28 (0.6)
r^2^ (p)	0.61 (0.1)	0.70 (0.2)
RR_10_/RR_50_/RR_99_	1.5/1.7/1.4	

WS- wild strain; LS – lab strain; LC – lethal concentration (ppm); RR- resistance ratio.

The results of insecticide susceptibility bio-assay for adult *St. albopicta* have been shown in Table 2. Corrected mortality (CM) obtained for DDT was 57.4 indicating that the mosquitoes were resistant to DDT. However, CM for deltamethrin and malathion was 98.8 and 98.6 respectively, which suggested that *St. albopicta* adult stage was fully susceptible to both of these insecticides. The knock-down values (KDT_10_, KDT_50_ and KDT_99_) obtained for DDT displayed straight line in log-dose-probit analysis and follow a linear regression model for knockdown with time (p = 0.9). The KRR_99_ for DDT was 4.9, indicating that there was a 4.9 fold increase in knock-down resistance to DDT. However, in case of malathion and deltamethrin, the KRR_99_ values were 1.6 and 1.5 respectively suggesting that mosquitoes were knock-down sensitive. In case of malathion and deltamethrin, the KRR_10_ values were 4.6 and 4.2 respectively and decreased thereafter for KRR_50_ and KRR_99_, indicating that mosquitoes had developed a certain level of tolerance to these insecticides which enabled them to survive the knock-down effect up to some time.

**Table 2:** **Mortality and knock-down in *St. albopicta *** **against the insecticides; 4% DDT, 0.05% deltamethrin (DM) and 5% malathion (MA)**

**Insecticide (N)**	**CM**	**KDT**_**10**_	**KDT**_**50**_	**KDT**_**99**_	**χ**^**2**^	**p**	**KRR**_**10/50/99**_
		**(95% CI)**	**(95% CI)**	**(95% CI)**			
DDT (143)	57.4	27.0	88.5	763.7	4.1	0.9	1.9/1.2/4.9
		(21.2-31.4)	(69.9-137.8)	(359.3-3429.4)			
DM (182)	98.8	3.8	9.4	48.0	16.8	0.002	4.2/2.1/1.5
		(1.6-4.4)	(6.0-12.3)	(42.8-131.4)			
MA (174)	98.6	28.4	74.1	422.0	94.3	0.0	4.6/3.2/1.6

CM - corrected mortality; KDT_10/50/99_ - time in minutes at which 10%/50%/99% knock-down achieved using log probit; KRR- knock-down resistance ratio.

Altogether 16 pools of female (N = 393) and 3 pools (N = 31) of adult male *St. albopicta* were tested for the presence of dengue virus in dipstick based strip assay but none of the pool was found positive for dengue virus.

## Discussion

During the last five decades, use of insecticides in agriculture as well as in public health has led to the development of resistance in mosquito vectors in many endemic countries [10,11,14,20]. In the present study, the larvae of the potential dengue vector *St. albopicta* were completely susceptible to the temephos and the LC values followed a normal linear distribution. The RR values at LC_50_ or LC_99_ were lower than 2, which are not considered biologically significant for resistance. The difference in RR values at LC_50_ or LC_99_ might be due to natural variations in toxicity ratios rather than to resistance selection [21]. Temephos is an organophosphate compound widely used as larvicide to control the vector mosquitoes. Recently, resistance to this insecticide has been reported in many Asian countries [12,22]. However, some studies have indicated that temephos is completely susceptible against wild *Ae. albopictus* and *Ae. aegypti* mosquitoes [10,14].

Adult bioassay results showed that the test mosquito species has developed resistance to DDT. Resistance to DDT might have been developed over time due to prolonged use in public health programmes for many years now. DDT resistance has been widely reported in *Aedes* mosquitoes worldwide [23,24], including India [14], however, the underlying mechanism still remains unclear. Although, the *kdr* mutation of voltage-gated sodium channel has been reported to confer resistance to DDT, but detoxifying enzyme activity can also play a vital role in the development of DDT resistance [23]. KDT values in the current study indicated that there was a high level (4.9 folds) of knock-down resistance in *St. albopicta*. Knock-down resistance is one of the most important forms of resistance related to the elevated detoxifying enzyme level in the mosquitoes.

The present study indicated complete susceptibility of *St. albopicta* mosquitoes to malathion and deltamethrin as the mortality obtained was above 98 percent. Malathion has been used mostly in fogging operations during the mosquito-borne diseases outbreaks and its activity is closely related to the acetylcholinesterase level of target mosquitoes. Pyrethroid resistance has been reported widely [11,23,24], and is associated with an altered amino acid sequence of the sodium channel and also through the elevated activity of detoxification genes. The KRR_99_ values of malathion and deltamethrin were below 2, suggesting that *St. albopicta* mosquitoes were knock-down sensitive. The KDT values obtained in both these insecticides did not display a normal distribution pattern. KDT values have been found to be associated with the level of use of insecticide [20]. No evidence of cross resistance to DDT and deltamethrin was observed in the current study, suggesting the involvement of metabolic resistance rather than a mutation in the sodium channel gene. In India, the data on insecticide resistance status of dengue vectors is scanty and therefore might be a limiting factor for the success in various control efforts. The intervention programmes are often recommended on the basis of evaluation at laboratory level but ignore the evaluation using wild mosquitoes from the field. Insecticide resistance is a dynamic phenomenon and even closely located populations could reflect variation in the insecticide susceptibility level.

Although none of the pool could be found positive for dengue virus, but this study is the first effort aimed at confirming identification of dengue vectors in Northeastern India. Many recent studies have detected the dengue virus in rural and urban settings of India and identified *St. albopicta* as a potential dengue vector [3,4]. A recent report on detecting dengue vector using Vector Test™ assay has suggested that the assay is highly specific and does not cross-react with other similar viruses [18]. The present study, probably due to the small sample size of *St. albopicta* mosquitoes used for detecting dengue virus was not able to identify dengue vectors but the present result cannot be generalised to the other areas where dengue outbreaks have been reported recently. Similar investigations using large numbers of possible dengue vectors in different endemic areas are urgently needed to identify potential dengue vectors.

## Conclusion

Wild collected *St. albopicta* was resistant to DDT and susceptible to temephos, malathion and deltamethrin. There was high level of knock-down resistance developed to DDT. Since dengue cases are rising annually, it is recommended that regular resistance surveillance should first be focused in areas of high dengue fever transmission in order to facilitate selection of insecticides with relatively higher assurance to minimise dengue infections. The identification of dengue vectors using easy and rapid assays and large sample size from different field areas could be useful to target the interventions.

## Competing interest

The authors declare that they have no competing interest.

## Authors’ contributions

SD and BR designed the experiments and performed the field study. SD, BR and KY performed the laboratory studies. SD and KY analysed the data and performed stastical analysis. SD and BR prepared the manuscript. IB and VV critically reviewed the manuscript. All the authors have read and approved the final manuscript.
